# Bioaccumulation of Cadmium Affects Development, Mating Behavior, and Fecundity in the Asian Corn Borer, *Ostrinia furnacalis*

**DOI:** 10.3390/insects11010007

**Published:** 2019-12-20

**Authors:** Mei Luo, Hong-Mei Cao, Ying-Ying Fan, Xiao-Cao Zhou, Jun-Xian Chen, Henry Chung, Hong-Yi Wei

**Affiliations:** 1 College of Agronomy, Jiangxi Agricultural University, Nanchang 330045, China; meiluo0522@126.com (M.L.); zxc_422@139.com (X.-C.Z.); cjx116799@163.com (J.-X.C.); 2 Department of Entomology, Michigan State University, East Lansing, MI 48864, USA; hwchung@msu.edu; 3 Jiangxi Sericulture and Tea Research Institute, Nanchang 330202, China; chm2017@163.com; 4 Geological Survey of Jiangxi Province, Nanchang 330030, China; fy6445952@163.com

**Keywords:** Asian Corn Borer, heavy metal, cadmium, development, mating, fecundity

## Abstract

Heavy metal pollution is becoming an increasingly serious problem in agricultural ecosystems. Heavy metals such as cadmium (Cd) accumulate in the food chain and may lead to detrimental effects on the physiological functions of living organisms, including herbivorous insects. One such example is the Asian Corn Borer, *Ostrinia furnacalis* (Lepidoptera: Pyralidae). However, how Cd can affect the development and reproduction of *O. furnacalis* is largely unknown. In this study, we exposed larvae of *O. furnacalis* to a diet containing Cd and investigated the effects of Cd on the development, mating behavior, and fecundity of the insect. We showed that Cd accumulates in the larvae and inhibits development by extending larval and pupal duration and decreasing the survival rate. The excretion of Cd through multiple routes during the larval and pupal stages resulted in low levels of residual Cd in the adult insects, which were not fed with Cd. However, the mating behavior and fecundity of these insects were significantly affected, compared to control insects. This suggests that the bioaccumulation of heavy metals such as Cd has long lasting and detrimental effects on *O. furnacalis* over the entire life cycle, affecting fecundity, even when specimens are only exposed at an early life stage.

## 1. Introduction

Heavy metal (HM) pollution in the environment results from natural phenomena, such as volcanic eruptions, and anthropogenic activities, such as fossil fuel burning, mining, industrial waste transpiration, and chemical fertilizer applications [1,2]. HMs accumulate in the water supply and soil, which are then transferred into plants, insects, animals, and humans via the food chain [3,4,5]. These HMs persist in organisms for a long time because they are not easily excreted by the organism. This eventually leads to high levels of HMs in the food chain [6,7,8].

HMs are toxic, even at low concentrations. An example of this is the HM cadmium (Cd) [9]. In recent years, Cd pollution has become increasingly worse, with numerous studies reporting increasing Cd pollution in water [10] and agricultural soil [11,12]. One serious consequence of Cd pollution is the accumulation of Cd in rice [13,14], a major food source for a large proportion of the world’s population [15]. The ingestion of Cd-contaminated rice can lead to cadmium poisoning and serious diseases, such as the “Itai-itai disease”, which causes bone softening and kidney failure in humans [16]. This suggests that Cd pollution has become a huge problem which does not just affect the environment, but also threatens food safety and human health.

In agricultural systems, plants persistently accumulate Cd from Cd-polluted soil and transfer Cd to higher trophic levels through the food chain [17]. Higher tropic-level consumers of crops, such as herbivorous insects, ingest Cd from their host plants and accumulate Cd [4,18]. For example, a continuous intake of Cd from plants leads to a high accumulation of Cd in the entire life cycle of hemimetabolous insects, such as the Chinese rice grasshopper, *Oxya chinensis* (Orthoptera: Acridoidea) [19]. In the large milkweed bug, *Oncopeltus fasciatus* (Heteroptera: Lygaeidae), continuous Cd exposure throughout the entire life cycle led to developmental defects and decreased reproduction [20]. Studies have also shown that Cd exposure has a negative effect on the growth and reproduction of holometabolous insects [21,22,23].

These studies mainly focused on the effects of continuous Cd exposure throughout the entire life cycle of these insects. However, in the field, insects such as Lepidopteran moths only feed on their host plants during the larval stage and do not feed again on the host plant during other life stages [24,25]. In addition, many migratory insects may only exhibit Cd intake during one life stage because of changes in their habitat [26]. Therefore, it is not known whether the accumulation of Cd during early life stages affects the development and reproduction of these insects throughout their entire life cycles. Understanding how Cd accumulates and the long-lasting effects of Cd, even when it has exited the organism, is of great importance as it underscores the seriousness of Cd pollution.

The Asian Corn Borer, *Ostrinia furnacalis* (Lepidoptera: Pyralidae), is one of the most destructive pests of corn in Asia, Australia, and the Solomon Islands [27,28]. With Cd pollution becoming a serious environmental problem in agro-ecosystems, including corn fields, *O. furnacalis* is constantly exposed to high levels of Cd in the environment [29,30]. However, there is little research regarding the effect of the accumulation of Cd on the development and reproduction of *O. furnacalis*. As a holometabolous herbivore, *O. furnacalis* only ingests Cd by feeding on its host plants during the larval life stage. The effects of accumulation at an early life stage on adult traits, such as mating behavior and fecundity, are largely unknown. As the sex pheromone system has been well-studied [31] and is sensitive to external stimuli [32], *O. furnacalis* can also be used as a suitable insect model to study the effects of Cd exposure at an early life stage on the mating behavior and fecundity in an adult life stage.

We exposed *O. furnacalis* to Cd at the larval stage and measured the accumulation and excretion of Cd throughout its entire life cycle. We also investigated the effects of the bioaccumulation of Cd on the development, mating behavior, and fecundity of *O. furnacalis* throughout the entire life cycle. In this study, we were interested in how *O. furnacalis* responds to Cd stress, and we specifically focused on the effects of Cd accumulation on mating behavior and fecundity in the adult stage of *O. furnacalis*, during which there is no Cd intake. Our previous research has studied the effects of Cd on the mating responses of female adults and showed that Cd stress can have detrimental effects on adult females, but we did not perform the experiment in males [33]. Therefore, in this study, we further measured the effects of Cd on mating responses of male adults.

## 2. Materials and Methods 

### 2.1. Insects and Rearing Conditions

The *O. furnacalis* laboratory colony was originally collected from field corn near the Jiangxi Agricultural University, Nanchang, Jiangxi province, China (28°46′05” N, 115°50′23” E), and was reared in a growth chamber for more than 10 generations. Insects were kept at 26 ± 1 °C with 70% relative humidity and a 14:10 h (L:D) photoperiod. An artificial diet was used for larval development in the laboratory [34]. Neonate larvae (hatched on the same day) were fed in plastic containers (15 cm diameter × 10 cm high) and mature larvae were transferred to boxes with straw paper to pupate. Pupae were sexed based on the morphology of the genital pore and oviposition opening of the female and kept separate before eclosion. Male and female moths were maintained separately in 50 × 50 × 50 cm screened cages housed and fed 10% sucrose water solution, as done previously [35].

### 2.2. Chemical and Reagent Doses

Cadmium chloride (Cd Cl_2_) was used in this study as the salt of heavy metal Cd. The salt was dissolved in distilled water to make a stock solution (2000 ppm). The stock solution was then diluted and added to the artificial diet to produce a series of artificial diets with different concentrations of Cd. Our previous study tested larval mortality rates in the different Cd treatments. We found that the larvae mortality rates were 17.5% (0.05 mg/kg), 20.1% (0.5 mg/kg), 22.5% (5 mg/kg), 31.4% (10.0 mg/kg), and 52.3% (20.0 mg/kg), separately. The baseline Cd content of the artificial diet was 0.031 ± 0.002 mg/kg. In the control treatment, no Cd was added to the artificial diet and the larval mortality rate was 6.3% [33]. A previous study has reported that the concentration of Cd ranges from 0.02 to 0.07 mg/kg in maize under different treatments [36], whilst it reached sublethal concentrations of Cd in *O. furnacalis.* In this study, three Cd treatments with three different artificial diets with lethal and sublethal concentrations of Cd (5, 10, and 20 mg/kg) were selected to test the Cd accumulations of different life stages. Due to a large number of insects being needed in the other experiments, *O. furnacalis* individuals were only fed an artificial diet with 5 mg/kg (sublethal dose) Cd in the Cd treatment. In this study, only larvae were fed Cd artificial diets, and there was no Cd input for pupae and adults.

### 2.3. Measurement of Cd Content

Thirty 5th instar larvae, two-day-old pupae, and two-day-old adults were randomly collected from different Cd treatment groups. Each treatment was replicated three to six times. All excretion products were collected from the 5 mg/kg Cd treatment groups and the control treatment groups. All 5th instar larvae samples were starved for 48 h to decrease the interference of undigested food in the determination of Cd content. The 5th instar larvae were transferred to boxes containing rough straw paper for pupation. We then collected larval exuvia, feces, and silk from these boxes where the pupa was kept, and collected pupal cases and wings after eclosion. Because the amounts of these excretion products were very small and were difficult to collect, we collected these in more than 10 different replicates. In Cd treatment, the control neonate larvae were fed a Cd artificial diet in each generation. All samples were collected and stored at −20 °C and then transferred to plastic petri dishes (10 cm in diameter) to oven-dry at 60 °C for over 36 h, until the body weight remained constant. The dried samples were weighed by an electronic balance to determine the weight of each larva, pupa, and adult sample. Each sample was then grinded into powder. The concentration of Cd was measured in the Geological Survey of Jiangxi Province, which is an attested institution for measuring the concentration of heavy metals. In total, 0.1–0.5 g powder samples were dissolved in 65% HNO_3_ solution until no solid material remained and the solution was clear. Each treatment was replicated three times. Five different concentrations of Cd^2+^ standard solutions were prepared for standard curves. The concentration of Cd in each sample was tested by Inductively Coupled Plasma Mass Spectrometry (ICP-MS) (7700, Agilent Technologies, Santa Clara, CA, USA). The ICP-MS parameters were as follows: RF power was set at 1550 W, plasma gas flow rate was set at 15.0 L/min, auxiliary gas flow rate was set at 0.9 L/min, and carrier gas flow rate was set at 1 L/min [37]. Cd concentration/individual (μg) = concentration (μg/g) * individual dry weight (g).

### 2.4. Developmental and Reproductive Evaluation

Neonate larvae that hatched on the same day were fed in plastic containers (15 cm diameter × 10 cm high) containing either an artificial diet with Cd (5 mg/kg) or control diets without Cd added. Two hundred 3rd instar larvae from these treatments were transferred to different new plastic containers for the observation of larval developmental duration. Fifth instar larvae were transferred to 24-well plates to pupate. All surviving larvae were used in the observation and calculation of the pupation rate. All pupae were used in the observation and calculation of pupal weights. Thirty paired adults of *O. furnacalis* were allowed to mate, including five paired adults, in the same transparent plastic bag and six replicates were performed. The number of eggs, and the egg hatchability of those eggs, were recorded in each group bag. During the experiment, the larvae duration, pupae duration, pupation rate, and emergence rate were also recorded. The weights of female and male pupae were measured with an electronic balance. Larvae duration was recorded from the date of larval hatching to the date of pupation. Pupae duration was recorded from the date of pupation to the date of emergence. Pupation rate = (the number of pupae/number of mature larvae) * 100%; emergence rate = (the number of adults/number of pupae) * 100%.

### 2.5. Mating Behavior Observation (Wind Tunnel Bioassay)

The behavioral responses of males were observed in a Plexiglas wind tunnel [38] in a darkened room (L14:D10 photoperiod, 26 ± 1 °C, relative humidity being 70%). The wind tunnel was cylindrical, with a length of 2 m, radius of 0.5 m, and wind speed of 0.3 m/s. This experiment was performed between 8 and 9 h after the initiation of scotophase (peak period of female calling).

Mating in *O. furnacalis* is initiated when a sexually mature female extends her pheromone gland in a process known as “calling” and releases a blend of sex pheromones to attract sexually mature males to mate. The male will then respond with a set of standard behaviors that were measurable in our assay. A previous study in our lab found that the calling frequency and calling duration were significantly reduced when females were exposed to Cd via diet [33] (Figure S1). To investigate whether Cd also affects mating behavior in males, we compared the mating responses of Cd-treated males and control males to untreated females.

One-day-old female adults from the control treatment were placed in a 4 cm^2^ mesh cage as the pheromone source and were placed 20 cm from the upper air outlet in all tests. The one-day-old males were released individually above the floor of the wind tunnel from a steel screen platform. The males were given 2 min to respond and the following behavioral categories were recorded: (1) TF: taking flight (male shaking of wings and taking flight); (2) OF: oriented flight (male-oriented flight to the female source); (3) HF: half up-wind flight (male-oriented flight half way to the female source); and (4) SC: source contact (male contact with the female source) [32]. Forty-one paired adults were tested in each treatment and each male and female were tested only once. The response time was from taking flight to source contact or stopping flight (did not contact source within 2 min). The times were recorded when males performed these four behavioral categories.

### 2.6. Statistical Analysis

Data were analyzed with SPSS (v24.0). The statistical significance of accumulation in different developmental stages and excretion products was determined by one-way ANOVA. Post hoc analyses were conducted using Tukey’s test at alpha = 0.05 if significant differences were found. The developmental time between Cd and control treatments was compared using the Mann–Whitney *U* test (non-normal distributions). The pupal weight, male response time, oviposition, and egg hatchability between Cd and control treatments were compared using Student’s *t*-test with normal distributions. Differences in the pupation rate, eclosion rate, and male behavioral responses were analyzed with a *Chi*-square test. Normality of the distribution was determined by a Shapiro–Wilk test (*n* < 50) and Kolmogorov–Smirnov test (*n* > 50).

## 3. Results

### 3.1. Multiple Routes of Excretion during the Life Cycle Result in Low Levels of Cd Residue in the Adult

**Figure 1:**
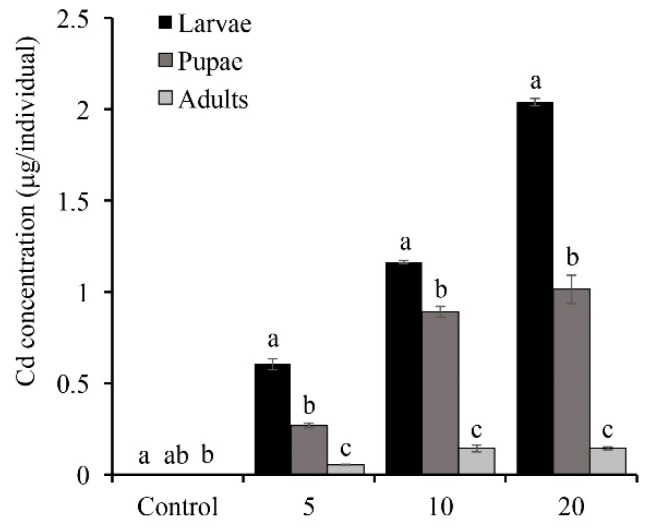
Cadmium (Cd) accumulation in different developmental stages of *Ostrinia furnacalis* by feeding on artificial food with three concentration of Cd. 5, 5 mg/kg Cd treatment; 10, 10 mg/kg Cd treatment; 20, 20 mg/kg Cd treatment. Cd content was measured by Inductively Coupled Plasma Mass Spectrometry (ICP-MS). Statistical analysis was performed by one-way analysis of variance followed by Tukey’s HSD test (α = 0.05). Bars and error bars are presented as the mean ± SE. The different letters in each treatment indicate a significant difference at a *p* < 0.05 level. *n* = 3–6.

We measured the bioaccumulation of Cd in the entire life cycle of *O. furnacalis*, where only larvae were fed on artificial food containing different concentrations of Cd. Larvae fed a 5, 10, and 20 mg/kg Cd diet showed the accumulation of 0.60, 1.16, and 2.04 μg Cd per 5th instar larva, respectively, and exhibited a dose-dependent response to Cd accumulation. We also found that larvae mainly accumulate Cd in the digestive tract (255.90 mg/kg), while the Cd concentrations in other tissues were very low (Table S1). Additionally, there was a gradual decrease in the Cd concentration from the larval stage to the pupal stage and the adult stage in all Cd treatments (Figure 1).

To determine the routes of Cd excretion in *O. furnacalis*, we measured the Cd contents in various excretion products in *O. furnacalis* fed with Cd (Table S2). We found that high levels of Cd were present in the feces (95.49 mg/kg), silk (82.98 mg/kg), pupal cases (76.87 mg/kg), and larval exuvia (25.88 mg/kg) in larvae fed with artificial food of 5 mg/kg Cd.

### 3.2. Cd Exposure in the Larval Stage Causes Detrimental Effects on the Development of O. furnacalis

**Figure 2:**
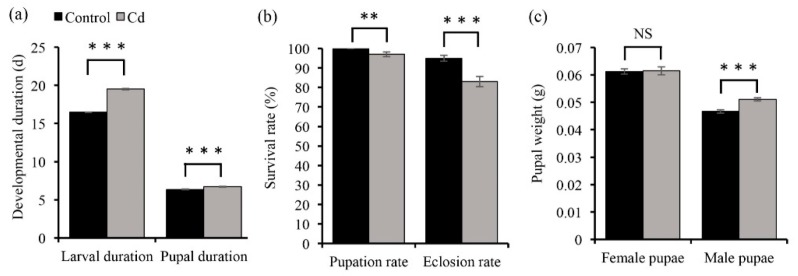
The effects of Cd on the (**a**) developmental duration, *n* (larvae) = 210, *n* (pupae) = 157–185; (**b**) survival rate, *n* (pupation) = 210, *n* (eclosion) = 192–201; and (**c**) pupal weight, *n* (male) = 82–86, *n* (female) = 106–119, of *O. furnacalis*. The developmental duration (Mann–Whitney *U* test), pupal weight (Student’s *t*-test), pupation rate (*Chi*-square test), and eclosion rate (*Chi*-square test) of Cd (5 mg/kg) treatment and control treatment were compared using different tests, according to the normality of distribution. Bars and error bars represent the mean and SE, respectively. NS, no significant effect; **, *p* < 0.01 and ***, *p* < 0.001, compared with the control treatment.

To determine if Cd affects the development of *O. furnacalis,* we compared the developmental timing, pupation rate, eclosion rate, and pupal weight of *O. furnacalis* fed with Cd and insects fed with a control diet (Figure 2). The results showed that larvae fed with Cd exhibited a significantly longer developmental time to pupation compared to control larvae (*p* < 0.001) (Figure 2a). Pupae also displayed a significantly longer developmental time to eclosion in the Cd treatment (*p* < 0.001). Cd caused a significant decrease in the pupation rate (*χ*^2^ *=* 7.12, *p* < 0.01) and eclosion rate (*χ*^2^ *=* 15.58, *p* < 0.001) in *O. furnacalis* (Figure 2b). Although the pupal weight was significantly decreased in male pupae under Cd stress (*t*_(219)_ = 5.22, *p* < 0.001), there was no difference in the female pupal weight between Cd fed individuals and control individuals (*t*_(191)_ = 0.13, *p* = 0.89) (Figure 2c).

### 3.3. Cd Exposure to Larvae Affects Adult Mating Behavior and Fecundity in O. furnacalis

**Figure 3:**
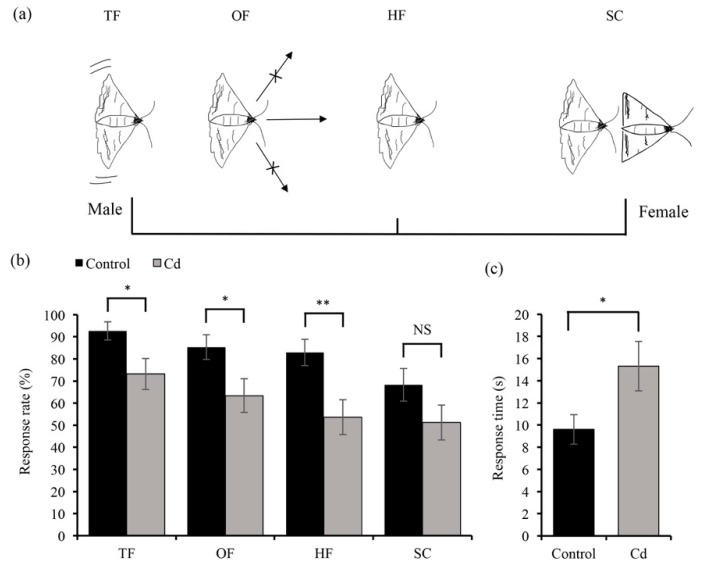
The effects of Cd stress on the mating behavior of male adults. (**a**) The mating behavior of male *O. furnacalis* in four stages. The four stages of male response to the female source, including TF, taking flight; OF, oriented flight; HF, half up-wind flight; and SC, source contact. (**b**) The response rate of males to the female source in these four stages. (**c**) The response time of males to the female source. The response time is from taking flight to source contact or stopping flight (did not contact source within 2 min). The response rate of males to the female source for Cd (5 mg/kg) treatment and control treatment was analyzed with a *Chi*-square test. Three males were non-responders in the control treatment and 11 males were non-responders in the Cd treatment. The response time of males to the female source for Cd and control treatments was compared using Student’s *t*-test. Bars and error bars represent the mean and SE, respectively. NS: no significant effect; *, *p* < 0.05 and **, *p* < 0.01, compared with control treatment. *n* = 22–41.

To determine whether Cd affects the mating behavior of *O. furnacalis* (Figure 3a), we compared the mating responses of Cd-treated males and control males to untreated females (Figure 3). The results showed that Cd-treated males displayed lower responses to female calling compared to the control males. Cd-treated males exhibited a reduction in behaviors such as ‘taking flight’ (*χ*^2^ *=* 5.51, *p* < 0.05), ‘oriented flight’ (*χ*^2^ *=* 5.19, *p* < 0.05), and ‘1/2 up-wind flight’ (*χ*^2^ *=* 8.11, *p* < 0.01), but there were no significant differences in the ‘source contact’ of female adults compared with control males (*χ*^2^ *=* 2.49, *p* = 0.12). Overall, Cd-treated males displayed significantly more time before contacting females than control males (*t*_(48)_ = 2.31, *p* < 0.05).

**Figure 4:**
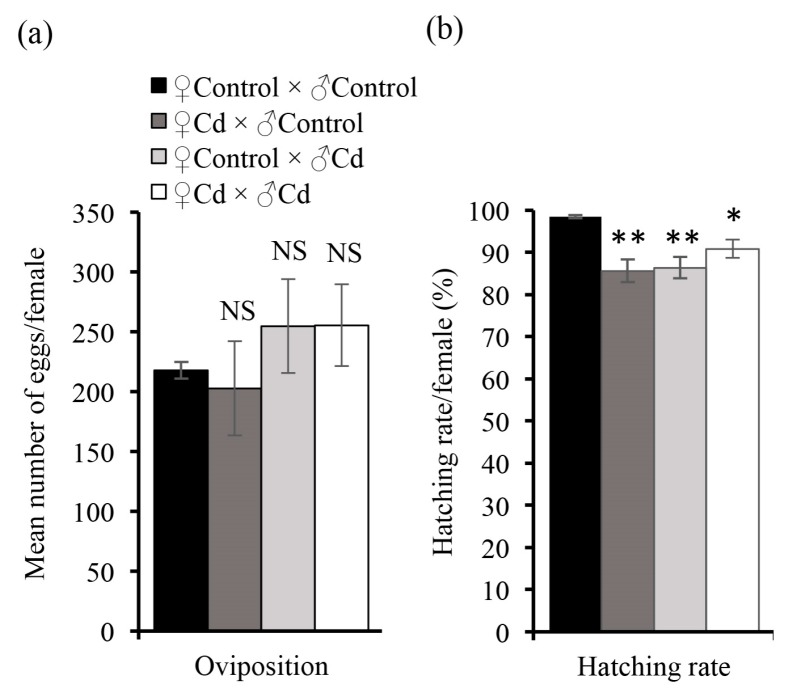
The effects of Cd on the oviposition (**a**) and egg hatchability (**b**) of *O. furnacalis*. The Cd (5 mg/kg) treatment and control treatment were compared using Student’s *t*-test. Bars and error bars represent the mean and SE, respectively. NS, no significant effect; *, *p* < 0.05 and **, *p* < 0.01, compared with control treatment. *n* = 6.

Fecundity in *O. furnacalis* was also affected by exposure to Cd. There were no differences in the oviposition of Cd females and control females (Figure 4a), but the egg hatching rate was significantly decreased, regardless of whether either parent or both parents were exposed to Cd (Figure 4b).

## 4. Discussion

A prolonged exposure of Cd exerts detrimental effects on living organisms due to its high degree of toxicity and rapid adsorption [2,22]. Insects ingest Cd from Cd-contaminated food and accumulate high concentrations of Cd in their body [19,23,39,40,41], which can suppress the growth and development of these insects [4,20,23,41]. However, many insect species do not continuously ingest Cd for their entire life cycle due to metamorphosis and changes in habitats. Such examples include holometabolous insects [24] and migratory insects [26].

In our study, we showed that in a holometabolous herbivore, *O. furnacalis*, Cd exposure in the larvae could have significant detrimental effects on development and mortality throughout the entire life cycle, even though there is no Cd intake in the pupal and adult stages. Previous studies have mainly focused on Cd residuals of feces and larval exuvia in the larval stage [39,42], which is the only life stage of host intake in the holometabolous herbivore. In our study, we also found that *O. furnacalis* could excrete high levels of Cd from silk and pupal cases in the late life stages. This leads to Cd being gradually excreted during the life cycle and low levels of Cd remain in the adults. However, these low levels of residual Cd also resulted in significant detrimental effects on the development and reproduction of adults.

In the present study, the male pupal weight of *O. furnacalis* was significantly higher for Cd treatment than control treatment, but the female pupal weight displayed no effect under Cd stress. In contrast to our study, Jiang et al. found that female/male pupal weights of *Lymantria dispar* (Coleoptera: Lymantriidae) were significantly decreased under Cd stress [43]. Similar reduction effects of Cd on body weight were reported in *Blaps polycresta* (Coleoptera: Tenebrionidae) [44]. The body weight of another insect species, *Spodoptera exigua* (Lepidoptera: Noctuidae), was not effected by Cd exposure [41]. Therefore, we suggest that the effects of Cd on pupal weight in insects differ among various species, and the effects vary between female and male individuals. We hypothesize that the different responses of body weight in different insect species may result from the different levels of Cd treatment.

Mating and fecundity are susceptible to toxicants such as insecticides, which can decrease the mating success in two different moth species [32,45]. Similarly, we previously found that Cd can affect mating in *O. furnacalis* by decreasing the calling frequency and calling duration of female adults [33] (Figure S1). In our present study, we showed that Cd can also affect mating in male *O. furnacalis* by decreasing the activation of male adults in courtship, and extending the response time of males, which may affect the mating success. Our results are similar to those presented in a recent study showing that Cd prolonged mating latency in another insect species, the vinegar fly *Drosophila melanogaster* (Diptera: Drosophiladae) [21]. This suggests that Cd could affect mating behavior, even in distantly insect species with different mating systems.

Cd stress did not affect the oviposition in *O. furnacalis*. However, a decrease of oviposition has been found in many insects exposed to Cd [20,43]. This suggests that Cd exposure has unclear effects on oviposition in insects. In our study, we also found that Cd exposure for either parent or both parents negatively affected egg hatchability in *O. furnacalis*, suggesting that gametic damage may occur in both sexes. Other HMs have been shown to affect fecundity by damaging gametes. Sun et al. reported that a high concentration of Ni damaged the quality of sperm bundles, resulting in a decrease of the oviposition and hatching rates in *Spodoptera litura* (Fabricius) (Lepidoptera: Noctuidae) [46]. Additionally, Osman et al. showed that Cd exposure caused general necrosis and shape change with distorted chromatin materials in oocytes, which damaged female gonads of the ground beetle, *Blaps polycresta* (Coleoptera: Tenebrionidae) [44].

Based on our results, we suggest that Cd exposure in larvae may result in gametic damage in both male and female adults, and the inhibition of mating behavior and fecundity of adults in *O. furnacalis*. Previous studies showed that the early formation, growth, and differentiation of egg formation begin in the larval stage [47] and Cd exposure could cause some general intracellular pathologies in insect cells [48,49]. We hypothesize that Cd exposure may cause irreversible damage in larvae cells, and this damage may also persist into adulthood, leading to gametic damage in adults.

## 5. Conclusions

In conclusion, we showed that Cd exposure during an early life stage has long-lasting detrimental effects on the development of *O. furnacalis* over the entire life cycle and could also result in adverse effects on mating behavior and fecundity, underscoring the urgent need to reduce Cd pollution in our shared environment.

## Supplementary Materials

The following are available online at https://www.mdpi.com/2075-4450/11/1/7/s1: Figure S1: The effects of Cd stress on the (a) calling frequency and (b) calling duration of female *O. furnacalis*; Table S1: Accumulation of Cd in different tissues and organs of larvae in *O. furnacalis* from Cd treatment (5 mg/kg); Table S2: The concentrations of Cd in different products.
