# Supplementary material for: Bioaccumulation of Cadmium Affects Development, Mating Behavior, and Fecundity in the Asian Corn Borer, Ostrinia furnacalis
Source: Insects. 2019 Dec 20;11(1):7. doi: 10.3390/insects11010007 (PMC7022320; doi:10.3390/insects11010007)
Supplement: Supplementary file 1 [file insects-11-00007-s001.docx]

**Supplementary Materials:** The following are available online at www.mdpi.com/xxx/s1, Figure S1: The effects of Cd stress on (a) calling frequency and (b) calling duration of female *O. furnacalis*, Table S1: The excretion of Cd in different excretion products.


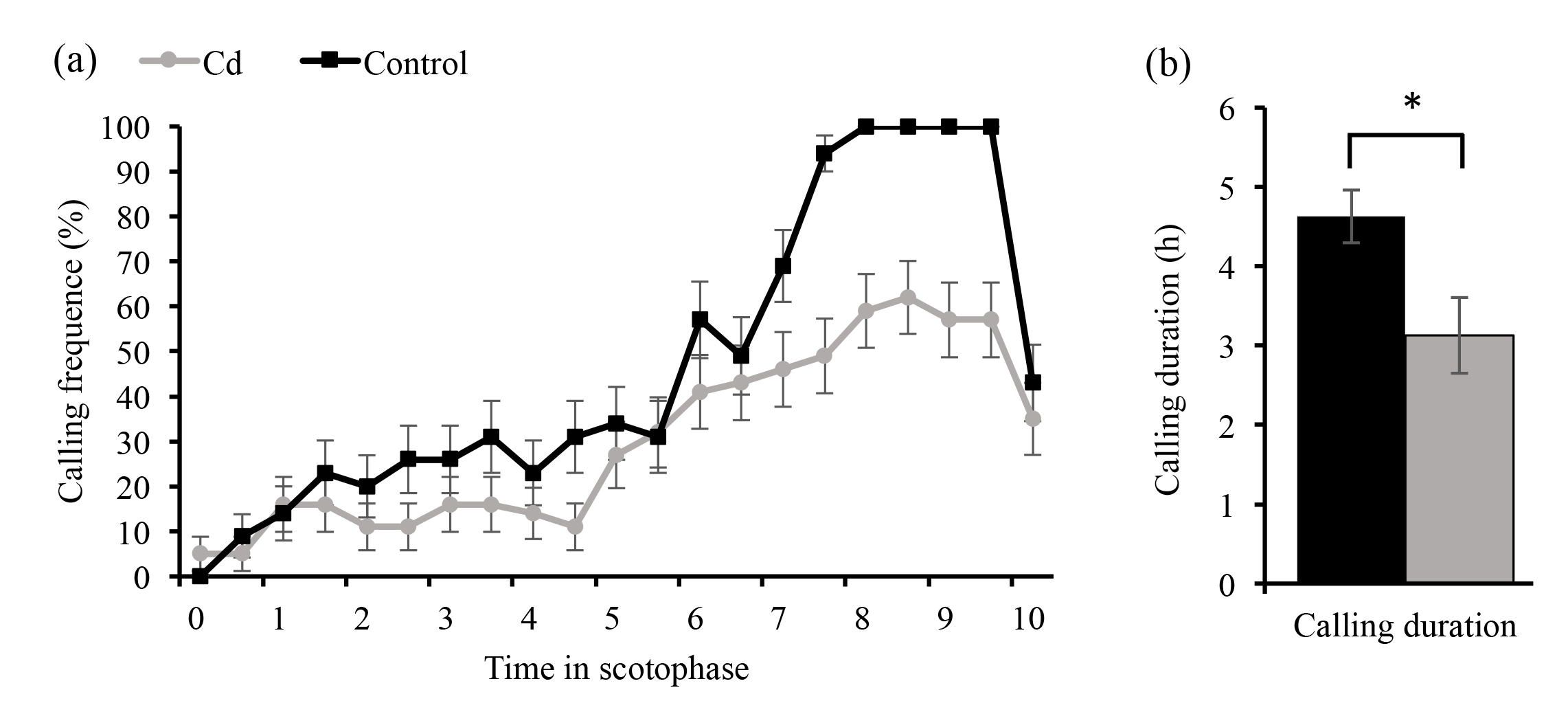


**Figure S1.** The effects of Cd stress on (a) calling frequency and (b) calling duration of female *O. furnacalis*. The calling duration in the Cd (5 mg/kg) treatment and control treatment were compared using Student’s *t*-test. Bars and error bars represent mean and SE respectively. *, *P* < 0.05 compared with control treatment.

**Table S1.** Accumulation of Cd in different tissues and organs of larvae in the *O. furnacalis* from Cd treatment (5mg/kg)

| Tissue or organ | Cd concentration/mg•kg-1 |
| --- | --- |
| Malpighian tubules | 8.0 ± 0.0 b |
| Digestive tract | 255.9 ± 0.6 a |
| Fat body | 1.8 ± 0.0 d |
| Cuticle | 1.0 ± 0.0 e |
| Head | 2.9 ± 0.0 c |

The Cd content was measured by ICP-MS assay. Statistical analysis was performed by analysis of variance followed by Tukey’s multiple range test (α = 0.05). The data are presented as mean ± SE. The different letters in each treatment indicated a significant difference at *P* < 0.05 level.

**Table S2.** The concentrations of Cd in different products.

| Product | Cd concentration/mg•kg-1 | |
| --- | --- | --- |
|  | Control | Cd (5mg/kg) |
| Feces | 0.7 ± 0.0 a | 95.5 ± 0.6 a |
| Silk | 0.7 ± 0.0 a | 83.0 ± 0.4 b |
| Pupal cases | 0.4 ± 0.0 b | 76.9 ± 4.2 b |
| Larval exuvia | 0.3 ± 0.0 c | 25.9 ± 0.2 c |
| Wings | 0.0 ± 0.0 d | 0.6 ± 0.0 d |

The Cd content was measured by ICP-MS assay. Statistical analysis was performed by analysis of variance followed by Tukey’s multiple range test (α = 0.05). The data are presented as mean ± SE. The different letters in each treatment indicated a significant difference at P < 0.05 level.
